# Antiphospholipid Syndrome in Renal Allograft Recipients—A Long-Term Multicenter Analysis

**DOI:** 10.3390/jcm12020667

**Published:** 2023-01-14

**Authors:** Agnieszka Furmańczyk-Zawiska, Barbara Bułło-Piontecka, Michał Komorniczak, Alicja Dębska-Ślizień, Hanna Augustyniak-Bartosik, Magdalena Durlik

**Affiliations:** 1 Department of Transplant Medicine, Nephrology and Internal Diseases, Medical University of Warsaw, Nowogrodzka 59, 02-006 Warsaw, Poland; 2 Department of Nephrology, Transplantology and Internal Diseases, Medical University of Gdańsk, Dębinki 7, 80-952 Gdańsk, Poland; 3 Department of Nephrology and Transplant Medicine, Medical University of Wrocław, Borowska 213, 50-556 Wrocław, Poland

**Keywords:** antiphospholipid syndrome, renal recipients, renal graft survival

## Abstract

Antiphospholipid syndrome (APS) is a devastating autoimmune disease and in renal transplant recipients may result in allograft thrombosis or in extra-renal manifestation, mostly venous thromboembolism. There are many non- and immune risk factors affecting renal allograft in recipients with APS. However, renal allograft outcome in recipients with APS without APS nephropathy remains unknown. Aim: The aim of the study was to assess renal allograft function and survival in recipients with APS. Methods: Retrospective, multicenter study included 19 adult renal recipients with definite APS (primary or lupus-related) from three Polish transplant centers. Renal allograft function was assessed using serum creatinine concentration (SCr1) at 3rd month post-transplant and at the end of the observation (SCr2) and glomerular filtration rate (GFR) was estimated based on modification of diet in renal disease (MDRD) formula. General linear model was used to assess 12 month GFR change over time. Kaplan-Meier curves and restricted mean survival time were used for allograft survival. Matched control group consisted of 21 stable renal recipients without history of thrombosis and without anticoagulation/antiplatelet treatment. Results: The study group differs in induction therapy (*p* = 0.019), high-urgency procedure (*p* = 0.04), proteinuria (*p* = 0.0058), primary disease (lupus) (*p* < 0.0001), re-transplantation in primary APS (*p* = 0.0046) and shorter time since engraftment to SCr2 (*p* = 0.016). Primary APS was more often diagnosed post-transplant (*p* = 0.0005). Allograft biopsy revealed thrombotic microangiopathy (TMA) with acute rejection (AR) or isolated AR vs AR or chronic rejection in controls but did not reach significance (*p* = 0.054). Renal allograft function was inferior in the study group but did not reach significance: mean SCr2 (mg/dL) was 2.18 ± 1.41 and 1.5 ± 0.68 in controls, respectively, *p* = 0.27; mean GFR2 (ml/min/1.73m^2^) was 39.9 ± 20.83 and 51.23 ± 19.03, respectively, *p* = 0.102. Renal allograft duration was inferior in patients with APS and was (in years) 11.22 ± 1.44 vs. 14.36 ± 0.42, respectively, *p* = 0.037, in patients with primary APS (*p* = 0.021), in patients with APS diagnosed post-transplant (*p* = 0.012) but not in lupus-related APS (*p* = ns). Fifteen year renal allograft survival was inferior in APS vs. controls (73,86% vs. 90.48%, respectively, *p* = 0.049). Conclusions: Recipients with APS are at higher risk for allograft loss due to immune and non-immune causes. Renal allograft survival was inferior in recipients with APS and renal function remains impaired but stable.

## 1. Background

Antiphospholipid syndrome (APS) is a devastating autoimmune condition characterized by recurrent venous/arterial thrombosis or pregnancy loss with persistent presence of heterogenous group of antiphospholipid antibodies (aPLs) that bind to protein-phospholipid complexes—anticardiolipin antibodies (aCL) and anti-β_2_-glycoprotein 1 (aβ_2_GP1)—while in a functional assay a group of lupus anticoagulant (LA) antibodies may be detected. There are three forms of APS—primary, associated with systemic disease (e.g., lupus-related APS) and a life threatening entity known as catastrophic APS (CAPS) with multi-organ failure and high mortality. Clinical presentation of APS includes a wide range of symptoms depending on type of organ/tissue involvement and expands from typical manifestation of thrombosis to severe CAPS.

APS nephropathy is a rare cause of end stage renal disease (ESRD) [1,2] and prevalence of renal involvement in APS is difficult to assess. In the literature, studies evaluated lupus patients with APS and, apart from lupus nephritis (LN), APS nephropathy was also frequently observed and was diagnosed in 63–67% [3,4]. Double or triple-positive patients for aPLs are at higher risk for renal involvement [5]. 

Renal involvement in APS consists of macrovascular complications (renal vein thrombosis, renal artery thrombosis/stenosis) and microvascular complication known as APS nephropathy affecting intrarenal blood vessels. 

APS nephropathy is defined as a non-inflammatory occlusion of intrarenal blood vessels (small vessel renal vasculopathy) and includes acute and chronic lesions. Acute form is represented by the presence of thrombotic microangiopathy (TMA) in glomerular capillaries with mesangiolysis and/or TMA in arteries and arterioles manifested by thrombotic lesions, intimal mucoid thickening and medial hyperplasia, while the chronic form of APS nephropathy is characterized by fibrous intimal hyperplasia, focal cortical atrophy and focal segmental glomerulosclerosis. Clinical manifestation of APS nephropathy is non-specific with hypertension (from moderate to malignant hypertension), sub-nephrotic range of proteinuria, hematuria, and acute kidney injury in 20–50%, but nephrotic syndrome is relatively rare [5,6,7].

In renal transplant recipients, clinical course of APS is unpredictable and thus may lead to allograft thrombosis and allograft loss. 

Prevalence and clinical impact of circulating aPLs in transplant recipients have been studied over time, whilst there are scarce data considering renal transplant recipients with definite APS and impact of this serious disease on renal allograft function and survival [8,9]. 

Aim: The aim of the study was to assess renal allograft function and renal allograft survival in recipients with primary APS and lupus-related APS. 

## 2. Material and Methods

This was an observational, multicenter, retrospective, cohort study consisting of 19 adult Caucasian renal transplant recipients from three Polish transplant centers. All recipients were treated with standard immunosuppressive regimen including prednisone, calcineurin inhibitor (mostly tacrolimus) and mycophenolate mofetil (MMF). Induction therapy including anti-Interleukin 2 receptor (anti-IL2R) agent was administered in medium risk patients, while thymoglobulin was offered in high risk patients (highly sensitized patient with panel reactive antibodies (PRA) > 80%, re-transplantation, living donation). The diagnosis of APS (primary or lupus-related) has been established in all enrolled recipients. Thromboembolic event was defined as an arterial/venous thrombosis or pregnancy loss or biopsy-proven TMA. A panel of aPLs including aCL and aβ_2_GP1 in IgG and IgM isotype were tested using the ELISA method, while LA was tested according to the recommendations of the International Society of Thrombosis and Hemostasis [10]. Renal allograft function was assessed using serum creatinine concentration (SCr) at 3rd month (SCr1) and at the end of observation (SCr2) an estimated glomerular filtration rate (eGFR) based on modification of diet in renal disease (MDRD) formula was calculated. Renal allograft biopsy was read by the same pathologist. Thromboprophylaxis consists of warfarin or low molecular weight heparin (LMWH) combined with low-dose aspirin when indicated. There was no allograft loss due to death. Mean observation time (in years) was 5.66 ± 4.90. Moreover, we identified three cases of CAPS in recipients with lupus-related APS. Due to their distinct clinical course, each case is described in Results.

Matched control group consisted of 21 renal transplant recipients with stable allograft function without thromboembolic complications and without antiplatelets/anticoagulation treatment. None of them have LN or APS as a primary native kidney disease. All the patients remain in post-transplant care at our institution. 

## 3. Statistical Analysis

Firstly, simple descriptive statistics were computed for all variables. Then, statistical association models were used to study the relationship between the studied variables and the response. 

During preliminary analysis, for qualitative variables, the hypotheses of independence were tested with chi2 test/Fisher’s exact tests according to the sample size. For quantitative variables, the hypotheses of the equality of distributions were tested with Wilcoxon Rank-Sum tests due to the lack of normal distributions of the variables.

Survival models were used to analyze time during the treatment processes. Times measured against events were subject to right-hand censorship. Therefore, the non-parametric Kaplan-Meier (KM) approach was chosen. The KM method allowed estimation of the probability of an event as a function of time based on individual data. The effect of risk factors on the length of time was investigated using log-rank tests. Additionally, Restricted Mean Survival Time (RMST) statistics were used as alternative tests. RMST is defined as the expected time to event value, calculated as the area under the survival curve to the end point. To obtain RMST, it was necessary to select a time point that clearly reflects the clinically relevant time horizon. 

Some analyzes were based on multivariate Generalized Linear Models (GLM). GLM allowed the testing of autocorrelated observations and sophisticated relationships between response variables and predictors. The Akaike Information Criterium (AIC) was used in the study as a goodness-of-fit statistic for selecting the optimal models. The REML estimation method was used in the calculations and the degrees of freedom in the significance tests were modified by the Satterthwaite method.

A *p*-value of <0.05 was considered statistically significant. The calculations were processed in SAS/STAT rel. 15.1. SAS Institute Inc., SAS Campus Drive, Cary, North Carolina 27513, USA. 

## 4. Results

Baseline characteristics of study group at transplantation are summarized in Table 1. 

Both groups were similar in terms of age, gender, number of transplant, type of donor (deceased/living), tolerance of immuno-suppression, number of human leucocyte antigen (HLA) mismatches, PRA-peak and current, type of donor specific antibodies (DSA) directed against HLA class I, II or both and time since engraftment to DSA detection, time since transplant to allograft biopsy, type of biopsy (protocol or for cause). Lupus nephritis as a primary disease was observed more often vs. other glomerulopathies diagnosed in the controls (*p* < 0.0001). 

High-urgency kidney transplantation (Ktx) was more often observed in the study group vs. controls (*p* = 0.0424) and induction therapy (anti-Il2R or thymoglobulin) was more often administered (*p* = 0.019); thymoglobulin was the preferred treatment in this indication (*p* = 0.013). The maintaining immuno-suppressive regimen including prednisone, tacrolimus and MMF was comparable (*p* = 0.056). Time (in months) since engraftment to graft biopsy (protocol or for cause) in the study group and controls was 35.33 ± 68.16 and 18.58 ± 21.07, respectively, *p* = ns. Graft biopsy revealed TMA with acute rejection (AR) or isolated AR vs. AR or chronic rejection (CR) in controls but did not reach significance (*p* = 0.054). Daily proteinuria > 1 g was frequently present (*p* = 0.0058). 

The study group consists of recipients with primary APS or lupus-related APS. The diagnosis of APS has been established prior to the 1st transplantation or post-transplant (Table 2). 

Analyzing renal allograft function we have found that SCr1 and GFR1 were comparable (Table 3); at the end of the study, worsening renal graft function was noticed but SCr2 and GFR2 did not reach statistical significance (mean SCr2 was 2.18 ± 1.41 vs. 1.5 ± 0.68 in controls, *p* = 0.27, mean GFR2 was 39.94 ± 20.83 vs. 51.23 ± 19.03, *p* = 0.102). Time since engraftment to the end of the observation was shorter in the study group (*p* = 0.016) (Table 3). 

**Figure 1:**
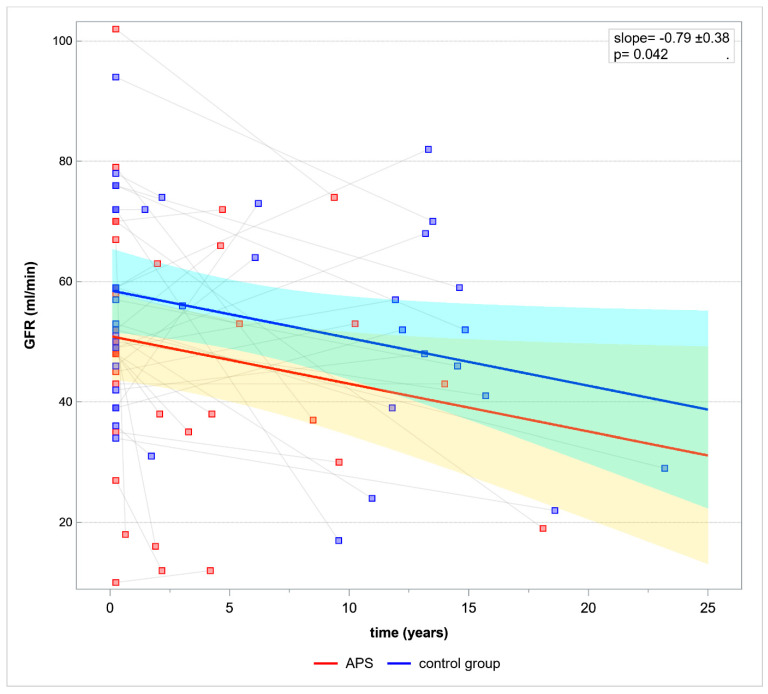
Model of GFR change over time. Abbreviation APS—antiphospholipid syndrome, GFR—glomerular filtration rate.

Based on GLM renal allograft function was evaluated in time. Mean GFR decline per year was 0.79 ± 0.38, *p* = 0.096, slope *p* = 0.042 (Figure 1).

**Figure 2:**
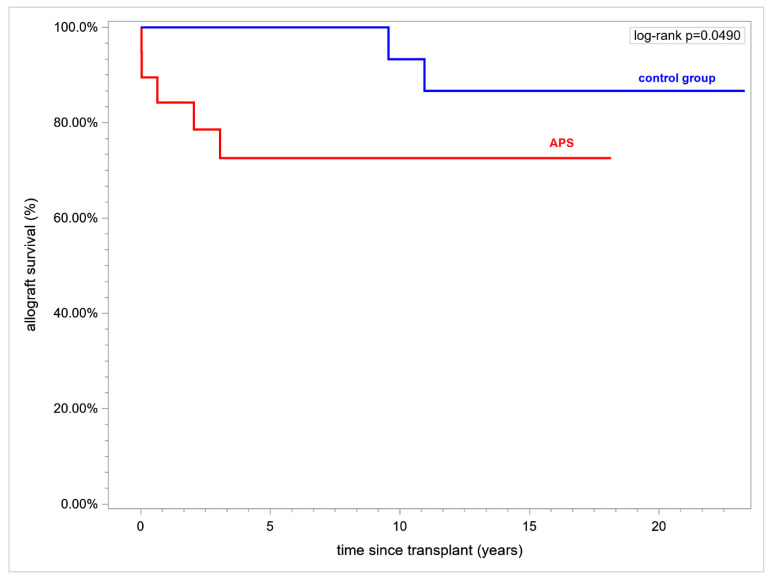
Kaplan-Meier curves. Renal allograft survival APS vs. controls. Abbreviation: APS—antiphospholipid syndrome.

Renal allograft duration over a time horizon of 15 years after transplant (RMST) was inferior in APS and was (mean ± standard error (SE), in years) 11.22 ± 1.44 vs. 14.36 ± 0.42 respectively, (*p* = 0.037). Fifteen year renal allograft survival was inferior in APS vs. controls (73.86% vs. 90.48%, respectively, *p* = 0.049) (Figure 2).

In addition, in all retransplanted patients, we have analyzed the first renal allograft survival over a time horizon of 15 years (RMST). The first allograft survival was significantly lower in patients with primary APS when compared to lupus-related APS at 0% vs. 75%, respectively. Allograft duration was also inferior in primary APS vs. lupus-related APS at 4.53 ± 2.3 vs. 11.33 ± 1.83 (in years, mean ± SE), respectively, *p* = 0.021. 

**Figure 3:**
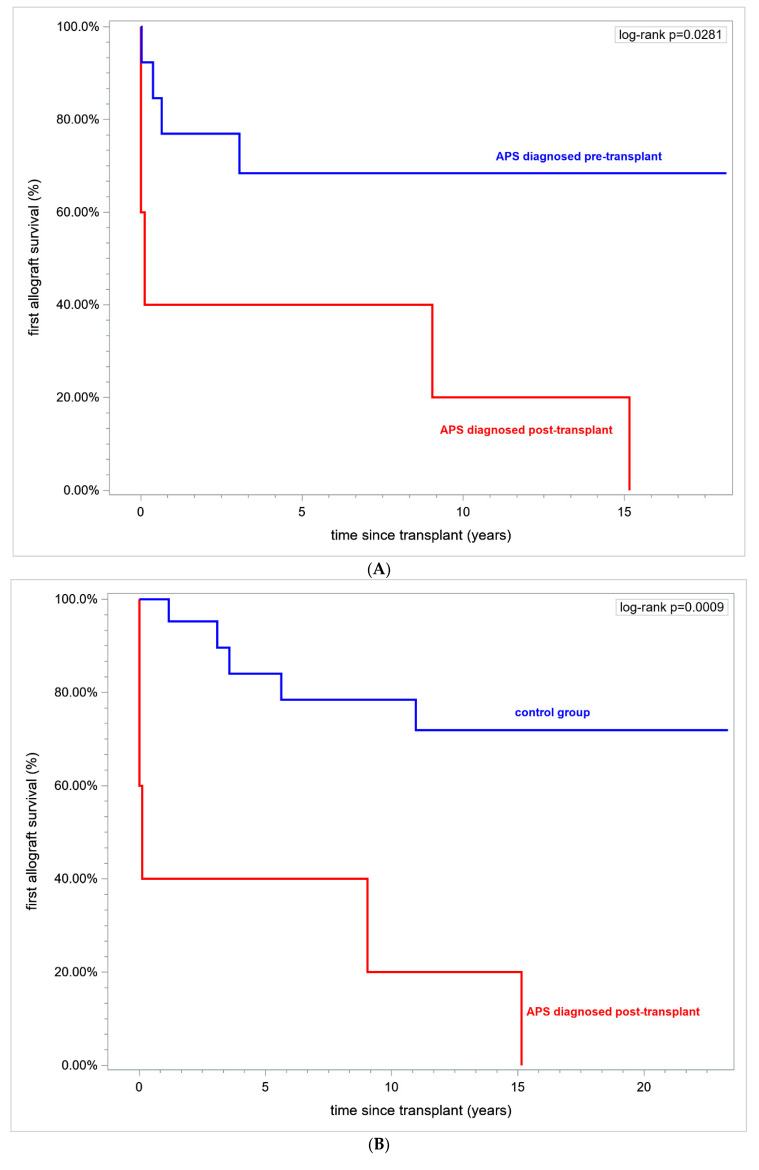

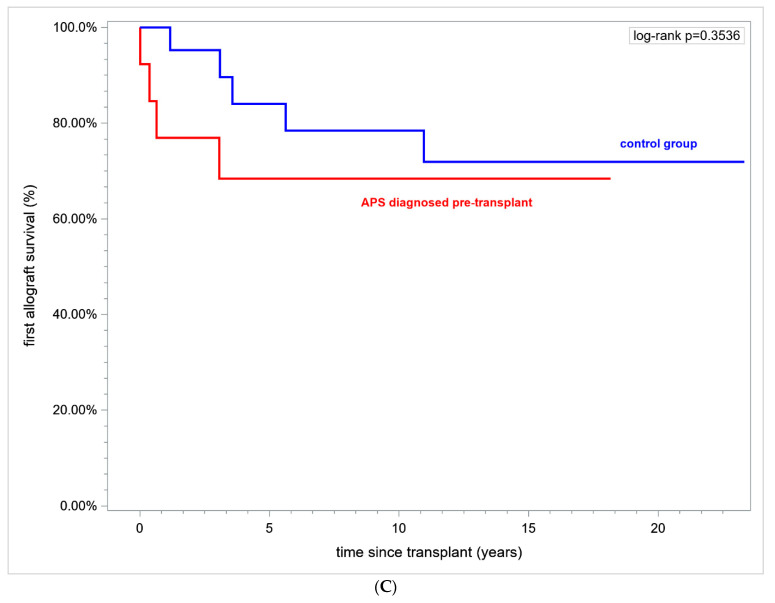
(**A**). Kaplan-Meier curves. First allograft survival in patients with APS diagnosed pre-transplant (prior to the first transplant) and APS diagnosed post-transplant. (**B**). Kaplan-Meier curves. First allograft survival in patients with APS diagnosed post-transplant vs. controls. (**C**). Kaplan-Meier curves. First allograft survival in patients with APS diagnosed pre-transplant vs. controls. Abbreviation: APS—antiphospholipid syndrome.

What is interesting is that, in patients with APS diagnosed post-transplant vs. patients with APS diagnosed pre-transplant, renal allograft survival was 0% vs. 69.23% and the first allograft duration was (in years) 4.83 ± 2.75 vs. 10.59 ± 1.83, respectively, *p* = 0.028 (logrank) (Figure 3A). 

In patients with APS diagnosed post-transplant vs. controls the first allograft survival was 0% vs. 76.19%, respectively, over a time horizon of 15 years and the first allograft duration in years (mean ± SE) was 4.83 ± 2.75 vs. 12.24 ± 1.11 in controls (*p* = 0.0126), (Figure 3B). 

Patients with definite APS prior to the first transplantation did not differ in renal allograft survival 69.23% vs. 76.19% (*p* = ns) and the first graft duration in years was 10.59 ± 1.83 vs. 12.24 ± 1.11 (mean ± SE), *p* = ns (Figure 3C). 

The study group consists of patients with primary APS and lupus-related APS. In 5/6 patients with primary APS the disease was diagnosed after transplantation—among these, 2/6 patients presented with deep venous thrombosis (DVT) complicated with pulmonary embolism (PE), and in the next 3/6 patients the disease was diagnosed as a result of early allograft thrombosis and allograft loss, therefore re-transplantation (second or third graft) was frequently observed (*p* = 0.0046). The past history of the study group revealed a wide range of thrombotic events such as an early allograft thrombosis resulting in allograft loss, TMA, PE and DVT, stroke, myocardial infarction in a young adult with no risk factors for cardiovascular complications, recurrent pregnancy loss, recurrent thrombosis of arterio-venous fistula > 3 episodes, thrombus in right ventricular (*p* = ns). 

Among the study group, primary APS was diagnosed in 31.58% and the rest of the group was diagnosed with lupus-related APS. The diagnosis of APS in patients with lupus was established prior to the first Ktx in each case, whilst the primary APS was more often diagnosed after the first Ktx (*p* = 0.0005) with severe clinical manifestation in allograft thrombosis. The secondary APS was due to lupus in all patients and LN was the cause of underlying native kidney disease. The pretransplant treatment of LN consisted of standard induction therapy including methylprednisolone and cyclophosphamide (class III and IV of LN) followed by maintenance therapy including prednisone and MMF. There were no signs of lupus flare at the time of transplantation and in follow-up lupus activity was low. In patients with primary APS, the end-stage renal disease (ESRD) was due to chronic glomerulonephritis, other than LN in four patients, autosomal dominant polycystic kidney disease (ADPKD) in one patient and renal calculi in some patients. The patients with lupus-related APS were frequently female (*p* = 0.03). Kidney re-transplantations (second or third transplant) were more common in patients who had not been diagnosed with APS prior to the first engraftment (*p* = 0.0046). 

When analyzing the primary and lupus-related APS, proteinuria was present in patients with lupus (*p* = 0.0006), but no differences were observed in renal allograft function at 3rd month and at the end of observation. 

Six episodes (31.56%) of recurrent thrombosis occurred in the study group in patients receiving thromboprophylaxis. Two patients with primary APS experienced graft TMA, two patients experienced stroke, one patient suffered from venous thromboembolism and in the last patient thrombosis of arterio-venous fistula occurred (*p* = ns). Anticoagulation treatment was complicated by bleeding in four out of six patients with recurrent thrombosis (Table 4).

In the study group there were five vs. two allograft losses in the controls during the observation time (*p* = 0.09). The cause of allograft loss was as follows: in the study group, an early graft thrombosis occurred in one patient (renal artery and vein thrombosis), TMA overlapping with CR in three patients, pure TMA not responding to treatment in one patient, while only episodes of CR were observed in controls. Among five allograft losses, four occurred in patients with APS diagnosed prior to the first Ktx, only one graft loss due to TMA, and CR occurred in one retransplanted patient with APS diagnosed post-transplant (*p* = ns). 

Monitoring of aPLs was applied in 15 patients (78.95%) as per center protocol. 

In our material, CAPS occurred in three recipients (two female) diagnosed with lupus-related APS prior to the first transplantation (data not shown). All of them were scheduled for quadruple immuno-suppressive treatment consisting of prednisone, tacrolimus, MMF and thymoglobulin. Renal allografts were harvested from cadaveric donor in two cases and in one case from a living donor. The first patient, a 31 year old male with persistent presence of ACL IgG treated with LMWH and aspirin, experienced an early allograft loss (within one week post-transplant) due to allograft thrombosis despite anticoagulation treatment. An early allograft thrombosis relapsed during his second renal transplant resulting in multiorgan failure. Post-transplant CAPS has been diagnosed, but due to thrombocytopenia and severe clinical condition renal allograft biopsy was not performed. Treatment based on methylprednisolone, therapeutic plasma exchange (TPE) and intravenous immunoglobulin (IVIG) infusion was administered immediately with no allograft improvement. Moreover, treatment was complicated by bleeding. Patient survived and remained dialysis-dependent. The next two patients experienced pretransplant CAPS with a severe clinical course responding to treatment (methylprednisolone, TPE, IVIG), but their post-transplant courses deserve attention. The second patient was a 32 year female with TMA, resulting in her first allograft loss at 3rd month post-transplant; at the time of her second transplantation from a living donor she was highly immunized (current PRA was 87%; historical PRA was 100%). She was triple positive for aPLs and received LMWH. Within 2 weeks post-transplant, no renal allograft improvement was observed; due to thrombocytopenia renal allograft biopsy was not performed and empirical treatment including methylprednisolone, TPE and IVIG was administered with no response. Patient was referred to dialysis. 

The third patent was a 47 year old female with a persistent double positive (LA, anti-β_2_GPI) aPL profile, treated with LMWH and aspirin. She was transplanted three times, her first and second renal graft post-transplant follow-ups were uneventful and late-onset graft losses were due to CR in both cases. Her third transplantation was complicated by slow graft function, renal graft biopsy excluded TMA and AR, and over the years renal graft function remained impaired but stable. 

## 5. Discussion

In clinical transplantology, the current approach toward renal recipients with circulating aPLs is associated with higher risk for renal allograft loss [9,11,12]. The prevalence of aPLs in transplant recipients is higher than in the general population [9,13,14]. Moreover, the underlying pathogenesis regarding how aPLs affect renal allograft remains unclear. [9,11,15]. However, the data comes from small allograft cohort studies. 

In this study, we evaluated renal transplant recipients with definite APS—primary or overlapping lupus; however, none of the patients had a diagnosis of APSN as an underlying native kidney disease. 

Renal transplant recipients with APS are at higher risk of allograft failure due to non-immune causes (graft thrombosis, ischemic-reperfusion injury, high urgency transplantation due to thrombosis of vascular access to hemodialysis) and immune causes (sensitization, re-transplantation, graftectomy, inferior HLA-matching due to high urgency transplantation, complications of induction treatment, episodes of antibody mediated rejection). Renal allograft function was inferior in the study group but it did not reach statistical significance probably due to a small sample size, similar to other authors’ observations [11,15]. Proteinuria is a recognized predictor of renal allograft loss [16]. The study showed that proteinuria not responding to treatment was significantly higher and represents an additional risk factor for deterioration of renal allograft function. Since APS has been diagnosed and anticoagulation treatment was introduced, renal allograft function remained impaired but stable. The most important findings of the study is that APS recipients. accumulating many immune and non-immune risk factors for poor allograft prognosis, continued to preserve renal allograft function. 

Much effort has been made in terms of long-term management of renal transplant recipients with APS, i.e., protocol biopsies, individualization of immuno-suppression and anticoagulation therapy and aPL monitoring. All of these circumstances allowed us to conclude that renal transplantation remains the best treatment option in APS patients regardless of the cause of ESRD. 

It is believed that APS has a negative impact on renal graft function and patient survival [15]. The vast majority of the previous studies analyzed allograft recipients with APS-nephropathy as a primary disease. Our study has demonstrated significantly lower allograft survival in patients with APS. 

In retransplanted, patients further analysis regarding the first renal allograft survival revealed significantly inferior outcome in the group of primary APS but not in the group of lupus-related APS. The explanation probably lies in a prevention strategy in the peri-transplant period. Each patient with APS diagnosed prior to the first transplantation was waitlisted with caution regarding thrombosis and given anticoagulation treatment as well as aPL monitoring, which could favor an uneventful post-transplant outcome. Lupus nephritis recurs rarely after transplantation. In our study, there was no lupus flare after transplantation as all recipients were given a triple immuno-suppressive regimen. 

On the other hand, our study shows that recipients with APS, despite anticoagulation treatment, experienced episodes of recurrent thrombosis-affected renal graft (two cases) as well as an extra-renal organ involvement were observed (four cases). In APS recipients, a higher rate of extra-renal thrombosis than allograft thrombosis has already been reported when compared to recipients with circulating aPLs without definite APS [11]. Based on our material, a similar observation was noticed and recurrence of thrombosis occurred in recipients persistently producing aPLs.

High-urgency transplantation and induction therapy were more often observed, similar to other authors’ observations [9] and, as mentioned above, this indicates that these patients are at higher risk for graft failure due to immune and non-immune causes. Time since engraftment to allograft biopsy was longer and demonstrated that this recognized gold standard procedure was performed reluctantly (risk of bleeding, risk of thrombosis when immobilized after biopsy). Some authors have proposed performing transvenous kidney biopsy in a patient at risk of bleeding [9] even in transplant recipients [17], but this time-consuming procedure requires more medical equipment and remains unavailable at our center.

The catastrophic form of APS is a rare, life threatening condition involving multiple organ failure with mortality rate near 50% and it may occur in renal transplant recipients as a severe relapse of APS, as a consequences of surgical procedure, trauma, anticoagulation withdrawal/change, infection, rejection and endothelial damage caused by immuno-suppression [6]. Treatment of CAPS remains a subject of debate, apart from that triple therapy (methylprednisolone, TPE, IVIG), rituximab or eculizumab may be promising but expensive options [18,19]. In our material, we identified two patients with a history of CAPS and one patient with post-transplant CAPS not responding to triple therapy and resulting in renal allograft loss. Unfortunately, in this case second-line treatment based on rituximab was contraindicated (infection) or unavailable in 2008 (eculizumab).

Recently published data show increasing interest in anti-C5 therapy in CAPS [20]. A linkage between circulating aPLs and complement activation resulting in complement-mediated TMA is known [21] but not fully understood. However, recently published data have shown that CAPS is associated with variants in complement genes similar to those observed in atypical hemolytic-uremic syndrome—mostly, complement factor H mutations are described [22,23]. This opens a discussion as to whether thrombotic APS or CAPS patient may benefit from eculizumab added to triple therapy, especially in refractory CAPS [21]. This promising approach raises a vexing question regarding duration of eculizumab treatment in such cases (consequences of chronic complement blockade, cost, frequent check-up every 2 weeks). Undoubtedly, this is a promising therapeutic modality and requires further study based on a larger sample. 

The major therapeutic goal is to prevent renal recipients from experiencing thrombosis and to maintain stable renal graft function. There are data showing that, despite adequate anticoagulation renal transplant, recipients with APS continued to develop vascular lesions resulting in graft deterioration, thus maintaining immuno-suppression based on inhibition of the mechanistic target of rapamycin (mTOR-i) complex pathway has been proposed [24]. This therapeutic approach with sirolimus provided protection against intimal hyperplasia and preserved renal graft function [6,24]. Recently, several studies have supported knowledge in the field of APSN, indicating that inhibition of mTOR complex is promising in recipients with APS [7,24,25]. Several years ago, inhibition of the mTOR pathway attracted a lot of attentions in terms of limiting nephrotoxicity of calcineurin inhibitor in transplant recipients. Nevertheless, many side effects have been reported since mTOR-i was introduced (delayed wound healing, proteinuria, severe pneumoniae, hyperlipidemia, diabetes, oedema and many others) which limited mTOR-i administration. In our study regarding immune status and risk of allograft loss due to immune causes, all recipients were scheduled to immuno-suppression based on calcineurin inhibitor. It must be underlined that none of the patients had biopsy-proven APSN as a primary native kidney disease. Nevertheless, considering the history of severe thrombosis shown in this material it is hard not to take into account that APS also plays an important role in this field. At this point, it is also important to mention that APS could be misdiagnosed or not treated properly. There are no guidelines that recommend screening in terms of aPLs prior to Ktx in all potential candidates. 

Anticoagulation therapy remains a challenge in thrombotic APS [26,27]. Recently published recommendations remind us of the use of unfractionated heparin in CAPS and to consider solid organ recipients. Anticoagulation is recommended before and after transplant; in case of invasive procedure (moderate to high risk of bleeding), oral anticoagulation should be suspended and bridging therapy based on heparin should be introduced while maintaining rigorous control [25,26]. In our study, all patients were anticoagulated with vitamin K antagonist (targeted INR 2–3) or LMWH adjusted to GFR and monitored with anti-Xa activity. Despite the anticoagulation treatment, thrombosis recurred six times during follow-up. In three patients, anticoagulation treatment was suboptimal due to bleeding complications, which seems to be the major obstacle in maintaining the treatment. Novel oral agents such as rivaroxaban gave conflicting results due to scarce data in APS [27,28] and currently remain contraindicated in triple positive APS and in arterial thrombosis [29]. 

Our study has several limitations. This is a retrospective analysis what may limit its findings. The small sample size is noticeable. Regarding the standard of aPL detection, in some patients with APS diagnosed many years ago it was not possible to compare the aPL profile at the time of APS diagnosis and at the time of transplantation. Thus, we based our report on aPL profile detected according to the current guidelines—i.e., collected in the peri-transplant period and during post-transplant follow-up. Moreover, recipients with overt thrombosis were enrolled to the study and probably had the most severe clinical course of APS, which also may bias the findings. 

## 6. Conclusions

Allograft recipients with APS are at higher risk of graft loss due to immune and non-immune causes. Fifteen year renal allograft survival in recipients with APS is significantly lower and renal allograft function remains impaired but stable over a long-term period. Considering the cost of Ktx, it is crucial to diagnose APS prior to Ktx. APl screening should be required in waitlisted candidates with lupus or history of thrombosis. Kidney transplantation remains the best treatment option in ESRD patients with APS.

## Figures and Tables

**Table 1:** Baseline characteristics of the patients.

Characteristic	Study Group *n* = 19	Controls *n* = 21	*p*-Value
Gender female/male	12/7	10/11	ns
Age (years) ± SD	41.5 ± 11.7	36.1 ± 13.8	ns
Number of graft (s)			ns
1st	11	17	
2nd	6	3	
3rd	2	1	
Mean observation time			
years ± SD	5.66 ± 4.90	11.17 ± 5.76	
Allograft loss	5	2	
High-urgency Ktx	4	0	0.04
Primary kidney disease			<0.0001
Lupus nephritis	13	0	
Chronic GN	4	13	
Nephrolithiasis	1	6	
PKD	1	2	
Donor origin deceased/living			ns
	16/3	19/2	
PRA median (range)			ns
current	0 (0–97)	0 (0–100)	
peak	7 (0–100)	3 (3–10)	
Number of HLA mismatches mean ± SD			ns
	4.2 ± 2.4	4 ± 2.4	
Induction treatment			0.019
Anti-Il-2receptor agent	5	2	
Polyclonal depleting globulin	10	4	
None	4	13	
		missing data in 2 individuals	
Maintenance IS			ns
Prednisone + tacrolimus + MMF	19	21	
IS tolerance			ns
good	13	14	
infection/diarrhea	5	5	
non-compliance	1	0	
Renal allograft biopsy			ns
protocol	1	11	
for cause	6	7	
Biopsy result			ns *p* = 0.054
TMA + AR	2	0	
AR	1	9	
Normal	4	9	
Daily proteinuria			*p* = 0.0058
<1 g	5	4	
≥1 g	8	1	
None	6	16	

Abbreviations: ns—not significant, Ktx—kidney transplantation, GN—glomerulonephritis, PKD polycystic kidney disease, PRA—panel reactive antibody, HLA—human leucocyte antigen, IS—immuno-suppression, MMF—mycophenolate mofetil, TMA—thrombotic microangiopathy, AR—acute rejection, SD—standard deviation.

**Table 2:** APS patient characteristics regarding type of APS (primary or lupus-related) and time of diagnosis (APS diagnosed prior to the 1st transplant or APS diagnosed post-transplant).

	Primary APS	Lupus-Related APS	Percentage
APS diagnosed pretransplant	1	13	73.64%
APS diagnosed posttransplant	5	0	26.32%
Percentage	31.58%	68.42%	

**Table 3:** Renal graft function.

Parameter	Study Group *n* = 19	Controls *n* = 21	*p*-Value
SCr_1_ (mg/dL) mean ± SD	1.55 ± 0.93	1.36 ± 0.36	*p* = 0.89
eGFR_1_ (mL/min/1.73 m^2^) mean ± SD	53.58 ± 20.92	56.33 ± 16.12	*p* = 0.62
SCr_2_ (mg/dL) mean ± SD	2.18 ± 1.41	1.5 ± 0.68	*p* = 0.27
eGFR_2_ (mL/min/1.73 m^2^) mean ± SD	39.94 ± 20.83	51.23 ± 19.03	*p* = 0.102
Time since engraftment to SCr_2_ (in months)	75.15 ± 58.31	134 ± 69.93	*p* = 0.016

Abbreviations: SCr_—_serum creatinine concentration, eGFR—estimated glomerular filtration rate.

**Table 4:** APS group characteristics.

Variables	Number of Patients (%)
APS (*n* = 19)	
primary (%)	6 (31.58%)
lupus-related (%)	13 (68.42%)
APS	*p* = 0.005
diagnosed prior to the 1st Ktx	14 (73.64%)
diagnosed post-transplant	5 (26.32%)
Type of thrombosis at the time of APS diagnosis	
graft thrombosis	5 (26.32%)
venous thromboembolism	9 (47.34%)
stroke/myocardial infarction	3 (15.78%)
avf thrombosis > 3x	1 (5.26%)
pregnancy loss	1 (5.26%)
Relapse of thrombosis on anticoagulation	5 (26.32%)
Type of thrombosis—relapse	
TMA	2 (10.52%)
Stroke	2 (10.26%)
DVT	1 (5.26%)
avf thrombosis	1 (5.26%)
Type of thrombo-prophylaxis	
VKA	10 (50.26%)
LMWH	5 (26.32%)
LMWH + ASA	4 (21.04%)
Complication of thrombo-prophylaxis	
Bleeding	5 (26.32%)
none	14
aPL monitoring	15 (78.95%)
Panel of aPL_1_	
Single positive	12 (63.15%)
LA	10
ACL IgG	2
Double positive	4 (21.04%)
Triple positive	3 (15.78%)
Panel of aPL_2_	8 (42.08%)
Single positive	5
LA	2
aβ_2_GP1 IgG	1
ACL IgM	1 (5.26%)
Double positive	2 (10.52%)
Triple positive–none or weak aCL	6 (31.56%)
	missing data in 2 individuals
CAPS	3 (15.79%)

Abbreviations: APS—antiphospholipid syndrome, Ktx—kidney transplantation, TMA—thrombotic micro-angiopathy, avf—arterio-venous fistula, VKA—vitamin K antagonist, LMWH—low-molecular weight heparin, ASA—aspirin, aPL—antiphospholipid antibody, aCL—anticardiolipin antibodies, CAPS—catastrophic APS, panel APLA: single positive (LA or aCl IgG), double positive (LA + aβ_2_GP1 IgG or aCl IgG or IgM), triple positive (strong LA + aβ_2_GP1 IgG + aCl IgG). Clinically relevant aPL titers include aCl IgG > 40 GPL, aCl IgM > 40 MPL, aβ_2_GP1 IgG or IgM > 99th percentile.

## Data Availability

No new data were created. This is retrospective study based on medical records.
